# A Red Blood Cell Membrane-Camouflaged Nanoparticle Counteracts Streptolysin *O*-Mediated Virulence Phenotypes of Invasive Group A *Streptococcus*

**DOI:** 10.3389/fphar.2017.00477

**Published:** 2017-07-18

**Authors:** Tamara Escajadillo, Joshua Olson, Brian T. Luk, Liangfang Zhang, Victor Nizet

**Affiliations:** ^1^Biomedical Sciences Graduate Program, University of California, San Diego, La Jolla CA, United States; ^2^Department of Pediatrics, Division of Host-Microbe Systems and Therapeutics, University of California, San Diego, La Jolla CA, United States; ^3^Department of NanoEngineering, University of California, San Diego, La Jolla CA, United States; ^4^Skaggs School of Pharmacy and Pharmaceutical Sciences, University of California, San Diego, La Jolla CA, United States

**Keywords:** *Streptococcus pyogenes*, streptolysin O, pore-forming toxin, neutrophil, macrophage, nanoparticle, biomimetic, antivirulence therapy

## Abstract

Group A *Streptococcus* (GAS), an important human-specific Gram-positive bacterial pathogen, is associated with a broad spectrum of disease, ranging from mild superficial infections such as pharyngitis and impetigo, to serious invasive infections including necrotizing fasciitis and streptococcal toxic shock syndrome. The GAS pore-forming streptolysin O (SLO) is a well characterized virulence factor produced by nearly all GAS clinical isolates. High level expression of SLO is epidemiologically linked to intercontinental dissemination of hypervirulent clonotypes and poor clinical outcomes. SLO can trigger macrophage and neutrophil cell death and/or the inactivation of immune cell functions, and promotes tissue injury and bacterial survival in animal models of infection. In the present work, we describe how the pharmacological presentation of red blood cell (RBC) derived biomimetic nanoparticles (“nanosponges”) can sequester SLO and block the ability of GAS to damage host cells, thereby preserving innate immune function and increasing bacterial clearance *in vitro* and *in vivo*. Nanosponge administration protected human neutrophils, macrophages, and keratinocytes against SLO-mediated cytotoxicity. This therapeutic intervention prevented SLO-induced macrophage apoptosis and increased neutrophil extracellular trap formation, allowing increased GAS killing by the respective phagocytic cell types. In a murine model of GAS necrotizing skin infection, local administration of the biomimetic nanosponges was associated with decreased lesion size and reduced bacterial colony-forming unit recovery. Utilization of a toxin decoy and capture platform that inactivates the secreted SLO before it contacts the host cell membrane, presents a novel virulence factor targeted strategy that could be a powerful adjunctive therapy in severe GAS infections where morbidity and mortality are high despite antibiotic treatment.

## Introduction

*Streptococcus pyogenes*, also known as group A *Streptococcus* (GAS), is a leading human-specific Gram-positive bacterial pathogen (Walker et al., 2014). GAS is responsible for significant disease morbidity and burden to the global healthcare system, producing an estimated 700 million cases of throat and skin infections annually. A clear increase in cases of severe invasive GAS infections, including sepsis, necrotizing fasciitis and toxic shock syndrome, has been documented in the last three or four decades, with mortality rates of 25% or higher (Wong and Stevens, 2013; Waddington et al., 2014). Coupled with its ability to trigger post-infectious immunologically mediated syndromes of glomerulonephritis and rheumatic heart disease, GAS ranks among the top 10 causes of infection-associated mortality in humans (Ralph and Carapetis, 2013).

The capacity of GAS to produce invasive human disease is the byproduct of a diverse array of bacterial virulence determinants that coordinately promote tissue invasion and resistance to innate immune clearance by host phagocytic cells including neutrophils and macrophages (Cole et al., 2011; Walker et al., 2014; Hamada et al., 2015; Dohrmann et al., 2016). These include the anti-opsonophagocytic surface-anchored M protein (Oehmcke et al., 2010) and hyaluronic acid capsule (Dale et al., 1996), resistant mechanisms against host defense peptides (LaRock and Nizet, 2015) and reactive oxygen species (Henningham et al., 2015), and secreted toxins capable of lysing phagocytes and/or disrupting their critical antimicrobial functions (Barnett et al., 2015).

Among the best studied GAS virulence factors is a potent secreted pore-forming toxin, streptolysin O (SLO). SLO is a cholesterol-dependent cytolysin that disrupts cytoplasmic membrane integrity of multiple eukaryotic cell types through pore formation (Tweten et al., 2015), thereby triggering cell death through apoptosis (Timmer et al., 2009), pyroptosis (Keyel et al., 2013) or programmed necrosis (Chandrasekaran and Caparon, 2016). SLO promotes GAS resistance to phagocyte killing (Sierig et al., 2003; Ato et al., 2008) and impairs critical phagocyte functions such as oxidative burst, migration, degranulation and neutrophil extracellular trap (NET) production (Uchiyama et al., 2015). Evolutionary genetic events associated with increased expression of SLO are associated with emergence of hypervirulent GAS clones and their rapid intercontinental dispersal (Zhu et al., 2015), as exemplified by the globally disseminated M1T1 clone that has emerged as the leading cause of severe, invasive infections in recent epidemiology (Aziz and Kotb, 2008; Nasser et al., 2014). Moreover, SLO expression is strongly upregulated by mutations in the *cov*R/S two-component transcriptional regulator that may arise *in vivo* and enhance systemic dissemination among M1T1 and other invasive GAS strains (Sumby et al., 2006; Cole et al., 2011). Mutation of the SLO gene or antibody-mediated inhibition of SLO toxin action is associated with reduced virulence in multiple murine models of invasive GAS infection (Limbago et al., 2000; Ikebe et al., 2009; Timmer et al., 2009; Chiarot et al., 2013; Uchiyama et al., 2015).

Anti-virulence strategies are gaining increased attention as a potential means to improve clinical outcomes in infections complicated by severe toxicity or antibiotic resistance (Clatworthy et al., 2007; Cegelski et al., 2008; Johnson and Abramovitch, 2017; Munguia and Nizet, 2017). Nanoparticle-based delivery systems have emerged as a key pharmacological platform for indications in diverse disease states including cancer (Schroeder et al., 2011) and diabetes (Sharma et al., 2015), and we and others have begun exploring their utility in counteracting bacterial toxin-mediated pathologies. Synthetic liposomal formulations can act as decoy targets for bacterial membrane-damaging toxins (Henry et al., 2015); however, a critical concern in nanotherapeutics is achievement of long circulation times to enhance clinical impact. Artificial nanocarriers may stimulate unwanted immunological responses or undergo relatively fast *in vivo* clearance (Luk and Zhang, 2015). Biomimetic nanotechnologies that utilize natural host cell membrane-coated nanoparticles may allay these concerns, bestowing stealth properties for increased circulation time and providing efficient interfacing to exploit known biological interactions (Kroll et al., 2017).

We have developed nanoparticles displaying red blood cells (RBC) membranes derived by hypotonic treatment and coated onto negatively charged poly-(D,L-lactide-*co*-glycolide) (PLGA) polymeric cores by extrusion or sonication methods (Kroll et al., 2017). These RBC membrane-camouflaged nanoparticles maintain right-side-out membrane orientation due to electrostatic repulsion with the PLGA core, remain stable in phosphate buffered solution as determined by polydispersity index (PDI) and the surface zeta potential, and possess an elimination half-life of ∼40 h before their clearance by hepatic macrophages without associated liver injury (Hu et al., 2011, 2014; Luk and Zhang, 2015). RBC membrane-camouflaged nanoparticles, or “nanosponges,” have been shown to act as decoy targets for purified versions of pore-forming toxins, including α-toxin produced by *Staphylococcus aureus*. Nanosponge administration sequestered α-toxin, rendering it harmless to mammalian cellular targets (Hu et al., 2013).

The current study takes our analysis of the pharmacological potential of RBC nanosponges to another leading pathogen, GAS, and its pore-forming toxin virulence factor, SLO. Employing wild-type (WT) and isogenic SLO-deficient mutant GAS strains, we assessed the effects of nanosponge administration upon SLO-mediated cytotoxicity and modulation of phagocyte antimicrobial functions in the context of live bacterial infection, and provide a first proof-of-principle of their therapeutic potential in a murine model of GAS necrotizing skin infection.

## Materials and Methods

### Bacterial Strains

Group A *Streptococcus* M1T1 serotype M1T1 5448 was originally isolated from a patient with necrotizing fasciitis and toxic shock syndrome (Chatellier et al., 2000) and an animal passaged (AP) version of the M1 5448 GAS parent strain (5448AP) containing a single inactivating adenine insertion at the 877-bp position of *covS* were used (Aziz et al., 2004). The isogenic M1T1 5448 ΔSLO mutant were described previously (Timmer et al., 2009). GAS strains were propagated using Todd Hewitt broth (THB) or agar (THA) at 37°C.

### Collection of Human Blood and Purification of RBCs or Neutrophils

Phlebotomy was performed on healthy donors with full informed consent under a protocol approved by the University of California San Diego (UCSD) Human Research Protections Program. RBCs were isolated for hemolysis assays or preparation of RBC nanosponges (see below). Neutrophils were isolated from freshly collected whole blood of healthy donors under a protocol approved by the, using PolyMorphPrep Kit (Fresenius Kabi) as previously described (Kristian et al., 2005).

### Mammalian Cell Culture

Human keratinocyte cell line HaCaT, murine macrophage cell line J774 and human monocyte cell line THP1 were cultured in RPMI-1640 media (Invitrogen) + 10% heat-inactivated fetal bovine serum (FBS) at 37°C in humidified air with 5% CO_2_. Primary bone marrow-derived macrophages (BMDM) were prepared as described (Hsu et al., 2004) with slight modification. Bone marrow cells were collected from mice and cultured in Dulbecco’s modified Eagle’s medium (high glucose) supplemented with 20% L-929 cell conditioned medium for 7 days. Adherent cells (BMDM) were then collected and cultured in Dulbecco’s modified Eagle’s medium (high glucose) with 10 ng/ml macrophage colony-stimulating factor (Pepro-Tech) overnight before bacterial infection.

### Generation of Human RBC-Derived Nanoparticles

RBC nanoparticles were prepared following published methods (Hu et al., 2011). Briefly, ∼100 nm PLGA polymeric cores were prepared using 0.67 dL/g of carboxy-terminated 50:50 poly-(D,L-lactide-co-glycolide) (LACTEL Absorbable Polymers) through a nanoprecipitation process. The PLGA polymer was first dissolved in acetone at a concentration of 10 mg/ml. One ml of the solution was then added rapidly to 3 ml of water, and the mixture placed in a vacuum for at least 3 h to evaporate the organic solvent. Human blood from healthy donors was washed with PBS + 1 mM EDTA × 3 by centrifugation at 500 × *g* for 10 min. RBC membrane vesicles were then prepared via hypotonic treatment and centrifugation at 10°C at 800 × *g* for 12 min. RBC membrane coating was completed by fusing RBC membrane vesicles with PLGA particles via sonication using an FS30D bath sonicator at frequency = 42 kHz and power = 100 W for 2 min.

### Bacterial Growth in Human Whole Blood

Blood was drawn from healthy donors after informed consent, and 2 × 10^5^ CFU of bacteria at OD_600_ = 0.4 were added to 400 μL heparinized whole blood in siliconized tubes. Tubes were placed on a rotator at 37°C for 1 h, then diluted and plated for colony forming unit (CFU) enumeration. Growth index was calculated as the ratio of surviving CFU after incubation vs. the initial inoculum.

### Macrophage and Neutrophil Killing Assays

J774 murine macrophages were seeded at ∼5 × 10^5^ cells in 350 μl of RPMI-2% FBS in a 24-well plate. Overnight bacterial cultures were diluted 1:10, subcultured for 3 h, resuspended and serially diluted in RPMI-2% FBS, and used to inoculate J774 cells at a multiplicity of infection (MOI) = 10 bacteria/cell. Freshly purified human neutrophils in serum-free RPMI were added to 96-well plates at a density of 1 × 10^6^ cells/well and infected with GAS at MOI = 1. Plates were centrifuged at 600 × *g* for 5 min to facilitate bacterial contact with cells. To assess total killing, macrophages were incubated at 37°C for 2 h and neutrophils for 15 min, washed three times with PBS, detached with 100 μl of 0.05% trypsin, and lysed with 900 μl of 0.025% Triton X-100 in PBS. Samples were serially diluted in PBS and plated on THA overnight for CFU enumeration.

### Cell Viability Assays

For quantification of cellular ATP as an indicator of metabolic activity and cell viability, opaque-walled 96-well plates with were prepared using THP-1 cells seeded at density 2 × 10^4^ cells/well, and infected with GAS WT or ΔSLO mutant strains at MOI = 25 for 2 h at 37°C. After incubation, 100 μl of CellTiter-Glo^®^ reagent was added to contents of wells, mixed for 2 min on an orbital shaker to induce cell lysis, and incubated at RT × 10 min to stabilize luminescent signal. Luminescence was recorded using SpectraMax plate reader and software. Live-Dead staining of HaCaT cells and murine BMDMs were performed by growing cells to 70% confluency in 96-well plates and infecting with WT GAS at MOI = 50 for 2 h, or 0.2 μg purified SLO for 30 min. Cells were then washed with PBS and treated with the viability assay mixture from the LIVE/DEAD Viability/Cytotoxicity Kit for mammalian cells (Molecular Probes, Invitrogen) for 30 min at 37°C, and imaged using an Olympus BX51 fluorescent microscope.

### Apoptosis Measurement

THP1 monocytes were plated at 1 × 10^6^ cells/well in 12-well plates and infected with GAS strains at MOI = 20:1. Plates were centrifuged at 2,000 rpm for 5 min to ensure bacterial contact with cells and then incubated at 37°C, 5% CO2. One hour after infection, penicillin (5 μg/ml) and gentamicin (100 μg/ml) were added to the media to kill residual extracellular bacteria. At 4 h after infection, cells were collected, fixed, and permeabilized for apo-bromodeoxyuridine TUNEL assay per manufacturer’s (BD Bioscience) instructions.

### Western Immunoblot Analysis

In a 6-well plate, 1 × 10^6^ J774 macrophages were seeded per well in 2 ml of RPMI + 2% FBS. Cells were infected with WT GAS at MOI = 10 for 2 and 4 h. To harvest whole-cell lysates, cells were washed three times with PBS and treated with radioimmunoprecipitation assay (RIPA) lysis buffer. Nuclear and cytoplasmic fractions were isolated using the NER-PER Extraction Kit (Pierse, Rockford, IL, United States) according to manufacturer’s protocol. Protein abundances were determined in cell fraction lysates with bicinchoninic acid assay (BCA) colorimetric assay. Aliquots containing 30 μg of protein were separated on 10% SDS-PAGE gels and transferred onto nitrocellulose membranes. Blots were probed using rabbit anti-Caspase 1 [Santa Cruz Technologies, diluted 1:500 in Tris-buffered saline–Tween 20 (TBST)]. Enhanced chemiluminescence reagent (PerkinElmer) was used for detection.

### IL-1β Measurement and Caspase-1 Activity Assays

J774 macrophages were infected at MOI = 10 for 2 h, supernatant collected and replaced with fresh media, then supernatant collected again 24 h post-infection. Skin from infected mice were homogenized in 1 ml PBS and centrifuged at 4°C, and the resulting supernatant used in ELISA for for IL-1β release (R&D Systems), via absorbance at 450 nm on a SpectraMax M3 plate reader and SoftMax Pro software. Caspase-1 activation was determined by Fam-YVAD-FMK (ImmunoChemistry Technologies) staining of THP-1 macrophages infected in 96-well plates per manufacturer’s specifications. Caspase-dependent apoptosis was determined in J774 murine macrophages using the APO-Caspase 3/7 activity assay (Promega) following the manufacturer’s protocol. Briefly, 100 μL of J774 cells were cultured in a white opaque 96-well plate at semi-confluency 1 day prior to infection with GAS at MOI = 20:1. Penicillin (5 μg/ml) and gentamicin (100 μg/ml) were added to the media 1 h after infection to kill residual extracellular bacteria. At 4 h after infection, caspase activity was quantified by adding 100 μL of detecting reagent per well, shaking gently for 5 min and incubating at room temperature × 1 h. Fluorescence at 520 nm was red on a Molecular Devices SpectraMax M3 reader to detect the level of caspase 3/7.

### Murine Infection Model

Mouse infections with GAS were performed based on modifications to a previously described model (Nizet et al., 2001) under a protocol approved by the UCSD Institutional Animal Care and Use Committee (IACUC). Twenty-four hours prior to infection, the backs of eight C57Bl6 mice were shaved and hair was removed by chemical depilation (Nair). Mice were infected subcutaneously with 50 μL of a sublethal dose of log-phase GAS (1 × 10^7^ CFU) in PBS, and 15 min later subsequently treated with vehicle only (10% sucrose) or vehicle plus 50 mg/kg of nanosponges in an area proximal to the site of the infection. Digital photographs of skin lesions were taken and lesion size was measured using NIH Imager software. Lesions were biopsied on day 3 post-infection. Excised lesions were placed into 2 ml screw cap tubes containing 1 ml PBS + 1 mm silica/zirconia beads (Biospec Products). Tissue was homogenized by shaking twice with the mini-beadbeater-8 (Biospec Products) at full for speed for 1 min, placing on ice in between. The homogenate was serially diluted in sterile PBS and plated on THA for enumeration. Dilutions were plated on THA agar and cultured overnight at 37°C for enumeration of CFU. Hematoxylin-and-eosin (H&E) staining was performed by the UCSD Histology Core Facility. Images were obtained using an Olympus BX41 microscope.

### Immunostaining of NETs and Elastase Release

Neutrophils (2 × 10^5^) were plated in 96 wells and infected with WT GAS and ΔSLO mutant at MOI = 1 in 37°C/5% CO_2_ for 4 h. Cells were fixed with 4% paraformaldehyde and stained with anti-myeloperoxidase (MPO) antibody (1:300 dilution, Calbiochem) in PBS + 2% bovine serum albumin (BSA, Sigma) at room temperature for 1 h, followed by incubation with goat anti-rabbit

Alexa 488 antibody (1:500 dilution, Life Technologies). Cells were counterstained with ProlongGold + 4’,6’-diamidino-2-phenylindole (DAPI, Invitrogen) and imaged on a fluorescent microscope. Representative, randomized images (*n* = 3) were taken for each condition and individualized experiment. Ratio of NET-releasing cells to non-NET releasing cells was determined as % of total cells. Elastase release from neutrophils infected with GAS at MOI = 5 for 30 min into the supernatant was indirectly determined using 20 μM peptide substrate *N*(Methoxysuccinyl)-Ala-Ala-Pro-Val 4-nitroanilide (Sigma) for 20 min at RT and absorbance 405 nm (SpectraMax M3 plate reader/SoftMax Pro). Statistical Analysis Experiments were performed in triplicate and repeated at least twice. Error data represent standard errors of the means (SEM) of the results from experimental duplicates, triplicates, or quadruplets. Statistical analysis was performed using Student’s unpaired two-tailed *t*-test. Comparisons among three or more samples were evaluated using one-way analysis of variance (ANOVA) followed by the non-parametric Tukey’s post-test. Comparisons of multiple samples were evaluated using ANOVA followed by Dunnett’s or Tukey’s test (Graph Pad Prism).

## Results

### RBC-Derived Nanosponges Block GAS SLO-Mediated Hemolysis and Keratinocyte Injury

**FIGURE 1:**
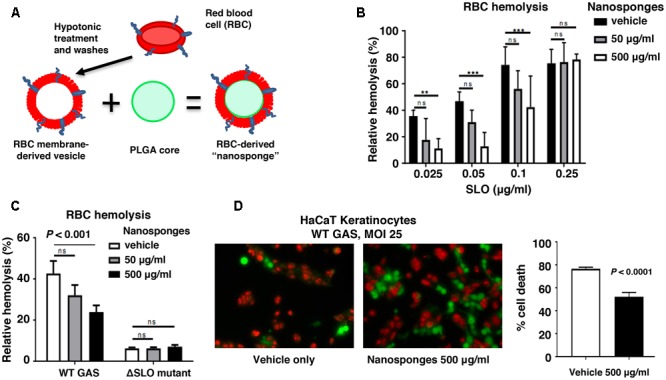
RBC nanosponges decrease GAS SLO-induced hemolysis and keratinocyte cytotoxicity. **(A)** Schematic showing fusion of RBC-derived ghost membrane vesicle and PLGA core to create nanosponge therapeutic. Human whole blood was centrifuged and washed with PBS + 1% EDTA to purify RBCs, then subjected to hypotonic treatment and centrifugation to rupture the membrane and remove intracellular content. PLGA cores were prepared from a 0.67 dL/g carboxy-terminated PLGA polymer by solvent displacement and resulting nanoparticles fused with RBC membranes by sonication. **(B)** RBC nanosponges inhibit purified SLO-induced RBC in a dose-dependent manner. **(C)** RBC nanosponges inhibit hemolysis induced by SLO produced by live WT GAS bacteria; isogenic SLO-deficient mutant serves as control. **(D)** Coincubation with RBC nanosponges increases survival of HaCaT keratinocytes infected with WT GAS at MOI = 25 bacteria/cell; live/dead cell staining illustrates viable (green) or dead (red) cells. Results are from experiments performed in triplicate, and reported as mean ± SEM from at least three experiments.

Creation of RBC-camouflaged nanoparticles involves two main steps: membrane vesicle derivation from RBCs followed by fusion of the membrane vesicle with the polymeric nanoparticle core (**Figure 1A**), as previously reported in detail (Desilets et al., 2001; Cheng et al., 2007; Hu et al., 2011; Pang et al., 2015). The RBC-derived nanoparticles, hereafter termed “nanosponges,” have an approximate particle size of 80 nm, right side out orientation, and other physical characteristics and physiochemical properties that lead to favorable pharmacokinetics and biodistribution (Hu et al., 2011). We determined that RBC nanosponges (50 or 500 μg/ml) could significantly inhibit the hemolytic action of purified SLO in a dose-dependent manner to an SLO concentration of 0.1 μg/ml (**Figure 1B**). Dot blot analysis (see Supplementary Methods) confirmed sequestration of SLO from the media to the nanosponges (Supplementary Figure S1). These findings mirrored prior observations with *S. aureus* α-toxin, in which RBC nanosponges absorbed α-toxin to limit its interaction with subsequent cellular targets, a finding that was absent when using just RBC membranes or polymeric cores individually (Hu et al., 2013). We then tested the ability of RBC-derived nanosponges to block hemolysis induced by coincubation of freshly isolated human RBC with a high level SLO-producing WT GAS strain (Supplementary Figure S2) compared to its isogenic ΔSLO mutant as a control. Paralleling results with the purified toxin, 500 μg/ml nanosponges produced a significant (∼50%) reduction of hemolysis induced by the WT strain, but had no effect on the low level of hemolysis seen in the ΔSLO mutant control (**Figure 1C**). SLO can also trigger membrane damage and cytolytic cell death in keratinocytes, which may play a role in the severe tissue injury of invasive GAS necrotizing skin infection (Ruiz et al., 1998; Sierig et al., 2003). As measured by LIVE/DEAD immunofluorescent staining, we found that administration of RBC nanosponges allowed a significant increase in survival of keratinocytes upon coincubation of the cells with the WT SLO-producing GAS strain at MOI = 25 (50% survival vs. 20% untreated, **Figure 1D**).

### RBC Nanosponges Counteract GAS SLO-Mediated Toxicity to Macrophages

**FIGURE 2:**
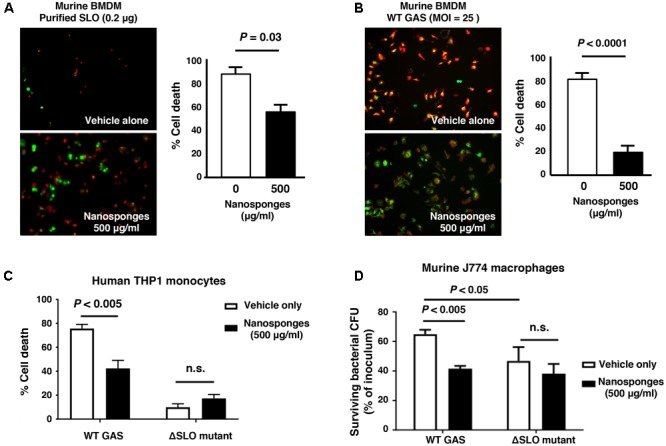
RBC nanosponges counteract GAS SLO-mediated toxicity to macrophages. RBC nanosponges significantly reduced cell death of murine BMDM following exposure to 0.2 μg purified SLO toxin **(A)** or infection with WT GAS at MOI = 25 bacteria/cell **(B)**; live/dead cell staining illustrates viable (green) or dead (red) cells. **(C)** RBC-derived nanosponges increased viability of human THP-1 macrophages exposed to WT GAS in a SLO-dependent manner. **(D)** RBC nanosponges enhanced murine macrophage killing of WT GAS to the level of killing observed with the isogenic GAS ΔSLO mutant. Results are from experiments performed in triplicate, and reported as mean ± SEM from at least three experiments.

**FIGURE 3:**
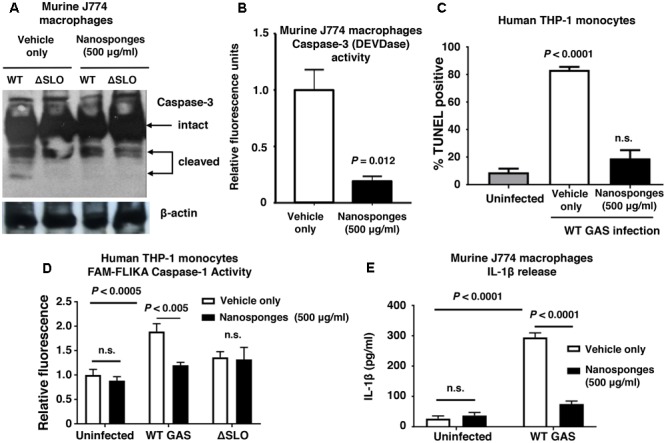
RBC nanosponges reduce GAS SLO-induced apoptosis and inflammasome activation in macrophages. Treatment with RBC-derived nanosponges reduced WT GAS induced macrophage apoptosis as measured by caspase-3 cleavage **(A)** and activity assays **(B)** performed in murine J774 macrophages, as well as TUNEL assay in human THP1 monocytes **(C)**. Treatment with RBC-derived nanosponges reduced GAS SLO-induced inflammasome activation measured by caspase-1 activity in human THP1 mononcytes **(D)** and IL-1β secretion in J774 macrophages **(E)**. Results are from experiments performed in triplicate, and reported as mean ± SEM from at least three experiments.

Macrophages contribute directly to bacterial clearance and produce cytokines and other inflammatory signals that can orchestrate downstream immune responses. As measured by live-dead staining, we found that RBC nanosponges significantly reduced cell death of murine BMDM following exposure to purified SLO toxin (**Figure 2A**) or WT GAS (**Figure 2B**). Similarly, RBC nanosponges increased viability of human THP-1 macrophages following incubation with GAS in a SLO-dependent manner (**Figure 2C**). RBC nanosponges also enhanced murine macrophage killing of WT GAS to the level of killing observed with the GAS ΔSLO mutant (**Figure 2D**). Treatment with nanosponges reduced WT GAS-induced macrophage apoptosis as measured by caspase-3 cleavage (**Figure 3A**), caspase-3 activity assay (**Figure 3B**), and TUNEL assay (**Figure 3C**). Nanosponge treatment also blocked GAS SLO-induced inflammasome activation (measured by caspase-1 activity, **Figure 3D**) and IL-1β secretion (**Figure 3E**). In sum, RBC nanosponges improved macrophage viability in the face of GAS SLO activation, enhancing their antibacterial function against the pathogen.

### RBC Nanosponges Mitigate GAS SLO-Mediated Impairment of Neutrophil Killing

**FIGURE 4:**
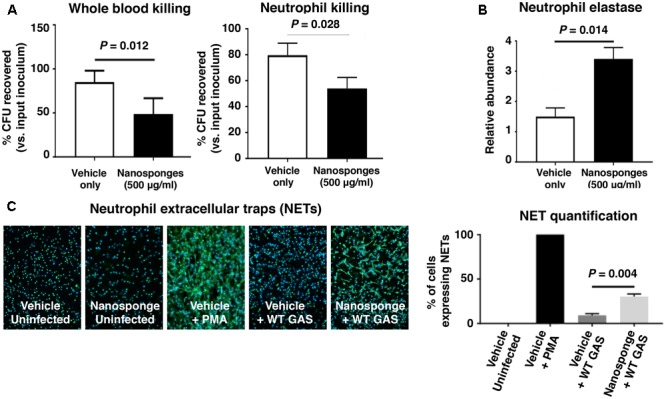
RBC nanosponges mitigates GAS SLO-mediated impairment of neutrophil killing. **(A)** Addition of 500 μg/ml of RBC nanosponges to freshly isolated human whole blood or purified human neutrophils significantly increases killing of WT GAS strain expressing SLO. **(B)** RBC nanosponge treatment increased neutrophil degranulation as measured by release of neutrophil elastase. **(C)** RBC-derived nanosponge treatment increased human neutrophil production of antibacterial NETs as detected by immunofluorescent staining and DNA quantification. Results are from experiments performed in triplicate, and reported as mean ± SEM from at least three experiments.

Group A Streptococcus production of SLO has been shown to impair neutrophil microbicidal activity, degranulation and release of DNA-based neutrophil extracellular traps, or NETs (Sierig et al., 2003; Ato et al., 2008; Uchiyama et al., 2015). We found that addition of 500 μg/ml of RBC nanosponges to freshly isolated human whole blood or purified human neutrophils significantly increased killing of the WT GAS strain expressing SLO (**Figure 4A**). Nanosponge treatment also increased neutrophil degranulation as measured by release of neutrophil elastase (**Figure 4B**), as well as the production of antibacterial NETs as detected by immunofluorescent staining and DNA quantification (**Figure 4C**). Coupled with the macrophage studies above, our *in vitro* analyses suggest that nanosponge sequestration of the GAS toxin can ameliorate damaging effects on innate immune phagocytes, allowing improved clearance of the pathogen.

### RBC Nanosponges Reduce GAS Disease Severity in Necrotizing Skin Infection Model

**FIGURE 5:**
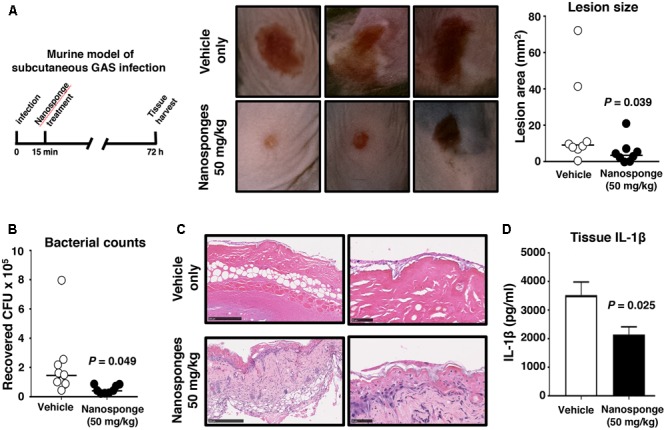
RBC nanosponges reduce GAS disease severity in necrotizing skin infection model. Invasive WT SLO-producing GAS strain subcutaneously into flanks of WT C57bl6 mice and mice were subsequently treated in the proximal tissues with either vehicle only control or vehicle + 50 mg/kg of RBC nanosponges (15 min post-infection). **(A)** At 72 h post-infection, necrotic skin lesions in the nanosponge-treated group were significantly smaller than those in the control group. **(B)** Quantitative bacterial cultures showed nanosponge-treated mice had significantly fewer GAS CFU recovered from wound tissue. **(C)** Representative histopathologic analysis of lesion biopsies showing reduced necrotic tissue injury in nanosponge-treated mice. **(D)** Levels of proinflammatory cytokine IL-1β produced in skin tissues in GAS-infected mice upon nanosponge administration. Statistical analyses performed using unpaired Student’s *t*-test. Results are shown as ±SEM.

Group A *Streptococcus* production of SLO has been shown to contribute to the severity of necrotizing skin lesions in murine experimental infection models (Limbago et al., 2000; Fontaine et al., 2003; Zhu et al., 2017). We injected the invasive WT SLO-producing GAS strain subcutaneously into the flanks of WT C57bl6 mice and then subsequently treated the mice proximally with either vehicle only control or vehicle + 50 mg/kg of RBC nanosponges at 15 min post-infection, a dose known to be well tolerated by mice in studies of purified α-toxin neutralization (Hu et al., 2013). At 72 h post-infection, necrotic skin lesions in the nanosponge-treated group were significantly smaller than those in the control group (**Figure 5A**), and quantitative bacterial cultures showed that nanosponge-treated mice had significantly fewer GAS CFU recovered from the wound tissue (**Figure 5B**). Histopathologic analysis of lesion biopsies showed wholescale hemorrhagic necrosis and tissue destruction of the dermal and subcutaneous tissues in control (vehicle-treated) mice; in contrast, nanosponge treatment preserved tissue architecture with markedly reduced necrotic changes and diminished neutrophil infiltration (**Figure 5C**). Furthermore, levels of proinflammatory cytokine IL-1β associated with inflammasome activation and pyroptotic cell death were reduced in the infected mice upon nanosponge administration (**Figure 5D**).

## Discussion

Group A *Streptococcus* infections continue to be a significant medical concern worldwide due in part to the high morbidity and mortality of severe invasive infections that require more aggressive care than antibiotics alone (Steer et al., 2008). Necrotizing fasciitis (aka “flesh-eating disease”) is an especially life-threatening form of invasive GAS infection that requires intensive supportive care and multiple modalities of treatment with limited established efficacy (Young et al., 2006). SLO is a critical GAS virulence factor linked epidemiologically and experimentally to tissue injury, resistance to immunological clearance and more severe pathology in necrotizing fasciitis and other forms of invasive GAS infection. Immune cell inhibition and destruction are major contributing factors to the progression of bacterial disease. Mice lacking macrophages or treated with inhibitors of macrophage phagocytosis cannot clear GAS infections even at low challenge doses (Goldmann et al., 2004), demonstrating their key front-line function in defense against the pathogen. GAS production of SLO damage macrophages (Ofek et al., 1972), including accelerating macrophage cell death pathways of apoptosis (Timmer et al., 2009) or oncosis (Goldmann et al., 2009). GAS expression of SLO may impair macrophage phagolysosomal fusion (Hakansson et al., 2005) and acidification (Bastiat-Sempe et al., 2014), facilitate GAS escape from the phagosome into the cytoplasm (O’Neill et al., 2016), block autophagic/xenophagic killing (O’Seaghdha and Wessels, 2013), or activate the NLRP3 inflammasome and IL-1β production/pyroptosis (Harder et al., 2009; Keyel et al., 2013). Here, we found that a biomimetic nanosponge constructed with a polymeric core wrapped in natural RBC bilayer membrane provided a substrate to absorb SLO, reduced its cytotoxic and immune inhibitory properties, promoted phagocyte clearance of GAS, and reduced disease pathology *in vivo* in a mouse necrotizing fasciitis model.

Neutralization of secreted toxins like SLO is an attractive anti-infective strategy, as it does not interfere directly with bacterial biochemical processes that exert selective pressure for antimicrobial resistance. Likewise, the antibiotic resistance profile of specific pathogen does not alter their susceptibility to toxin neutralization. An additional advantage of the anti-virulence nanosponge therapeutic is high specificity to target only the pathogenic infection, without deleterious effects on the normal host microbiome inherent in conventional broad-spectrum antibiotic therapy. In principle, the biomimetic RBC membrane shell provides substrate mimicry of a human host cell target capable of absorbing a wide range of pore-forming toxins of GAS and other pathogens regardless of their molecular structures.

Our proof-of-principle studies indicate that RBC nanosponges can counteract multiple pathogenic processes attributed to SLO and that their local administration can reduce bacterial burden and disease progression in an *in vivo* model of GAS necrotizing fasciitis. These experiments suggest there is merit in expanded analysis of the detailed pharmacokinetic and pharmacodynamic properties of this nanotherapeutic platform to expand investigations to multiple models of invasive infection with GAS and other pathogens in which disease outcome is driven in significant part by the deleterious effects of secreted membrane-active toxins.

## Author Contributions

LZ and VN conceived of the study. All authors designed experiments. TE, JO, and BL performed experiments. All authors analyzed data. TE and VN drafted manuscript which all authors critically reviewed.

## Conflict of Interest Statement

The authors declare that the researchwas conducted in the absence of any commercial or financial relationships that could be construed as a potential conflict of interest.
