# Supplementary material for: A Red Blood Cell Membrane-Camouflaged Nanoparticle Counteracts Streptolysin O-Mediated Virulence Phenotypes of Invasive Group A Streptococcus
Source: Front Pharmacol. 2017 Jul 18;8:477. doi: 10.3389/fphar.2017.00477 (PMC5513932; doi:10.3389/fphar.2017.00477)
Supplement: Supplementary file 1 [file Data_Sheet_1.PDF]

Supplementary Materials

A red blood cell membrane-camouflaged nanoparticle counteracts streptolysin O-mediated virulence phenotypes of invasive group A *Streptococcus*

Tamara Escajadillo, Joshua Olson, Brian T. Luk, Liangfang Zhang, Victor Nizet

Contents:

Supplementary Methods

Supplementary Figures S1 and S2 with Legends

Supplementary Methods

Dot blot analysis

Control (10% sucrose) or nanosponges were incubated in 1.5 ml Eppendorf tubes in the presence or absence of purified streptolysin O (SLO) + 10 mM dithiothreitol (DTT). Samples were incubated at 37°C for 30 min, centrifuged at 2000 rpm for 5 min, supernatants transferred to new tubes, and the pellet re-suspended in PBS. Samples were dropped in 5 µl aliquots onto a nitrocellulose membrane and dried completely before blocking with 5% milk x 1 h. The membrane was washed x 3 with Tris-buffered saline + 0.1% tween (TBST), incubated with anti-SLO antibody (ABCAM ab188539) at 1:1000 for 16 h, washed with TBST x 3, secondary rabbit antibody added for 1 h, washed x 3 with TBST, and enhanced chemiluminescence reagent (PerkinElmer) was used for detection. Images were quantified using jPEG imaging software.

RBC Hemolysis Assay

Supernatants from bacterial cultures collected at OD_{600} = 0.4 were used to assay hemolysis activity of released SLO. Purified SLO toxin activity was assessed using 10 mM dithiothreitol (DTT) to stabilize the toxin. Assays were allowed to proceed for 30 min at 37°C, with PBS (0% hemolysis) and 0.025% Triton (100% hemolysis) as negative and positive controls, respectively. Supernatants were collected from assay wells after centrifugation at 3000 x *g* for 10 min, and hemolysis determined by absorbance with SpectraMax M3 plate reader at 541 nm using SoftMax Pro software. Each titer was recorded as the point where hemolysis reached half of the 100% RBC lysis (0.025% Triton) control.

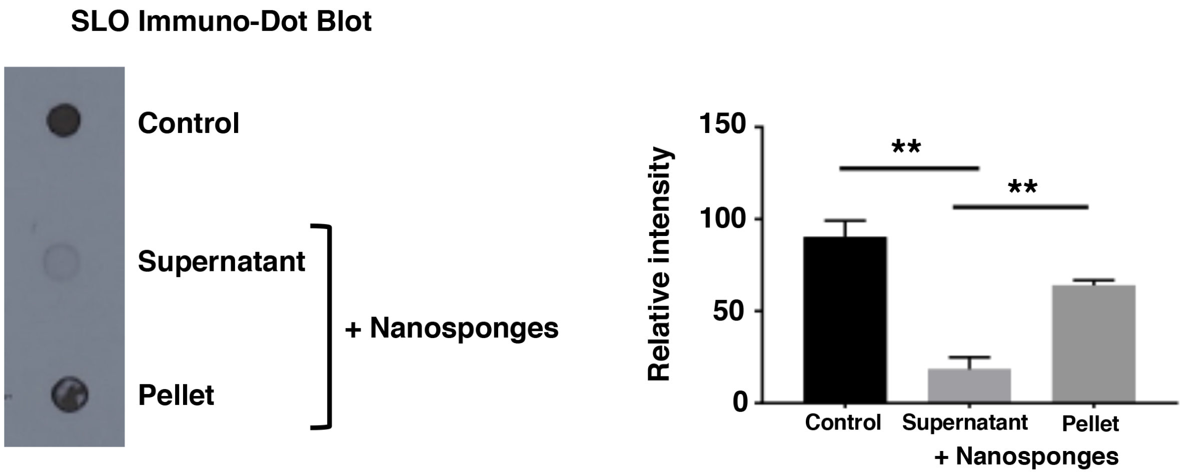

Supplementary Figure S1 | RBC nanosponges absorb SLO toxin from solution. Vehicle alone (10% sucrose) or 500 μg/ml nanosponges suspended in vehicle were incubated in 1.5 ml Eppendorf tubes in the presence of purified streptolysin O (SLO) + 10 mM dithiothreitol (DTT). Samples were incubated at 37°C for 30 min, centrifuged at 2,000 rpm, and supernatants and pellet collected and resuspended in PBS. 5 μl aliquots were dropped onto a nitrocellulose membrane for immunoblot detection using an anti-SLO antibody and enhanced chemiluminescence quantified using jPEG imaging software.

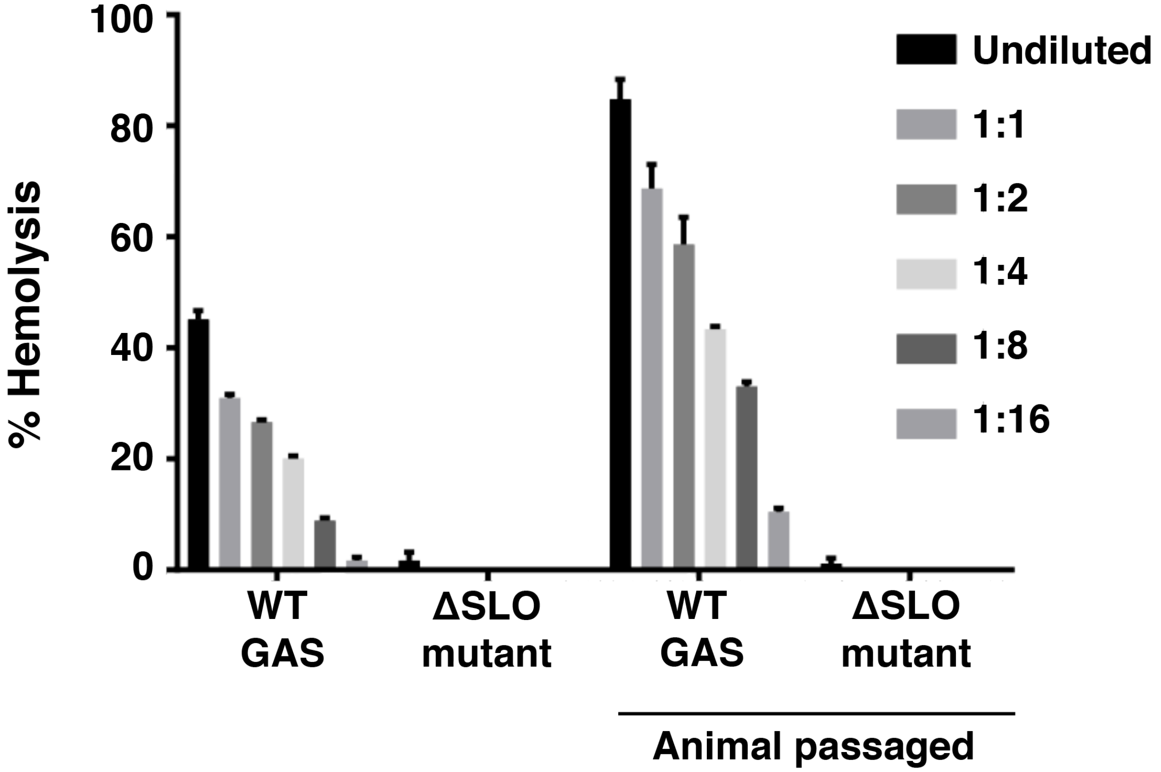

Supplementary Figure S2 | RBC nanosponges reduce GAS SLO-induced apoptosis and inflammasome activation in macrophages. Supernatants from bacterial cultures collected at OD_{600} = 0.4 were used to assay hemolysis activity of released SLO using 10 mM dithiothreitol (DTT) to stabilize the toxin. Assays proceeded for 30 min at 37°C, with PBS (0% hemolysis) and 0.025% Triton (100% hemolysis) as negative and positive controls, respectively. Supernatants were collected from assay wells 3000 x *g* centrifugation x 10 min, and hemolysis assayed by absorbance at 541 nm. Each titer was recorded as the point where hemolysis reached half of the 100% RBC lysis (0.025% Triton) control.
